# A Marked Point Process Framework for Extracellular Electrical Potentials

**DOI:** 10.3389/fnsys.2017.00095

**Published:** 2017-12-18

**Authors:** Carlos A. Loza, Michael S. Okun, José C. Príncipe

**Affiliations:** ^1^Department of Mathematics, Universidad San Francisco de Quito, Quito, Ecuador; ^2^Department of Neurology and Center for Movement Disorders and Neurorestoration, University of Florida, Gainesville, FL, United States; ^3^Computational NeuroEngineering Lab, Electrical and Computer Engineering Department, University of Florida, Gainesville, FL, United States

**Keywords:** electrocoticogram (ECoG), electroencephalogram (EEG), local field potentials (LFP), marked point process, robust processing, transient model, unsupervised learning

## Abstract

Neuromodulations are an important component of extracellular electrical potentials (EEP), such as the Electroencephalogram (EEG), Electrocorticogram (ECoG) and Local Field Potentials (LFP). This spatially temporal organized multi-frequency transient (phasic) activity reflects the multiscale spatiotemporal synchronization of neuronal populations in response to external stimuli or internal physiological processes. We propose a novel generative statistical model of a single EEP channel, where the collected signal is regarded as the noisy addition of reoccurring, multi-frequency phasic events over time. One of the main advantages of the proposed framework is the exceptional temporal resolution in the time location of the EEP phasic events, e.g., up to the sampling period utilized in the data collection. Therefore, this allows for the first time a description of neuromodulation in EEPs as a Marked Point Process (MPP), represented by their amplitude, center frequency, duration, and time of occurrence. The generative model for the multi-frequency phasic events exploits sparseness and involves a shift-invariant implementation of the clustering technique known as k-means. The cost function incorporates a robust estimation component based on correntropy to mitigate the outliers caused by the inherent noise in the EEP. Lastly, the background EEP activity is explicitly modeled as the non-sparse component of the collected signal to further improve the delineation of the multi-frequency phasic events in time. The framework is validated using two publicly available datasets: the DREAMS sleep spindles database and one of the Brain-Computer Interface (BCI) competition datasets. The results achieve benchmark performance and provide novel quantitative descriptions based on power, event rates and timing in order to assess behavioral correlates beyond the classical power spectrum-based analysis. This opens the possibility for a unifying point process framework of multiscale brain activity where simultaneous recordings of EEP and the underlying single neuron spike activity can be integrated and regarded as marked and simple point processes, respectively.

## 1. Introduction

Extracellular electrical potentials (EEP) from the brain can be recorded at distinct levels—from cm^2^-scale non-invasive electroencephalographic signals from the scalp (EEG) to invasive mm^2^-scale cortical activity recorded by subdural grid electrodes (ECoG) or even at localized deeper anatomical brain regions by inserting electrodes or silicon probes into the brain (LFP) (Buzsáki et al., 2012). This type of activity reflects the average spatiotemporal interaction of neuronal assemblies and, therefore, constitute a coarser scale (mesoscopic) measure beyond single-cell recordings (action potentials or spikes) that can play a complementary role to relate multiscale brain activity to more overt types of cognitive phenomena and psychological constructs, such as behavior, perception and learning. Moreover, these extracellular electrical potentials have been thoroughly studied in both clinical and research fields and further associated to complex processes, such as sleep (Rechtschaffen et al., 1968; Borbély et al., 1981), epilepsy (Nakasatp et al., 1994; Iasemidis et al., 2003), Parkinsons's disease (Soikkeli et al., 1991; Handojoseno et al., 2015), distributed perception (Gray et al., 1989; Tallon-Baudry and Bertrand, 1999), memory consolidation, spatial navigation and cognition (Llinas and Ribary, 1993; Kahana et al., 2001; Buzsáki, 2002), and stimuli processing by top-down influences (Engel et al., 2001).

Neuromodulations or phasic events in the EEP are the direct result of spatiotemporal synchronization of local neuronal populations in the form of distinctive, organized, reoccurring, transient (phasic) patterns that differ from the noisy featureless background in EEP, which is known to display a 1/*f* power spectrum (Freeman, 1975; Buzsaki, 2006). These phasic events have been well documented in the literature under the concepts of induced potentials and event-related oscillations (Tallon-Baudry and Bertrand, 1999; Freeman and Quiroga, 2012), e.g., gamma oscillations in the olfactory bulb of cats and rabbits after odor presentation (Freeman, 1975), characteristic sleep-stage-related patterns in humans (Rechtschaffen et al., 1968) and sharp-wave ripples in the hippocampus associated to cognition and memory processes (Buzsáki, 2015). The concept of transient events has been throughly echoed in the literature (Rechtschaffen et al., 1968; Nakasatp et al., 1994; Friston, 2000; Hopfield and Brody, 2001; Freeman and Rogers, 2002; Freeman et al., 2003); specifically, brain dynamics constantly shift between complex and predictable states by means of synchronization and oscillations (Buzsaki, 2006). Moreover, there is a growing clinical interest in Parkinsons's disease and synchronized transient events that could explain the freezing of gate phenomenon and other related mechanisms (Hammond et al., 2007; Lewis and Barker, 2009). Unlike evoked potentials, another well-known type of organized activity, event-related oscillations are characterized by latency and onset variability, which demand for fine temporal resolution and special attention when it comes to processing and further interpretation.

An important goal is to discriminate in time the phasic events (that resemble wave packets) from the temporally disorganized but spatially structured background activity, which appears as “noise” for the signal processing algorithms applied to each lead. This task is particularly challenging when considering the specific dynamics and statistical properties EEP recordings display, e.g., correlated colored noise, non-linear generation mechanisms, and non-stationarity. Early in the history of computer-based EEG analysis, there were two competing methods (Niedermeyer and da Silva, 2005): the time based quantification methods that used the properties of the EEP in the time domain, such as Hjorth parameters (Hjorth, 1970) or zero crossing analysis (Gaillard, 1987), and anthropomimetic methods that attempted to quantify the events electroencephalograhers recognized by eye balling on the strip charts for clinical applications (Smith et al., 1975). The latter were used to recognize epileptic spikes, K-complexes, spindles in the alpha, sigma, beta and gamma bands, and runs of theta and delta waves. Moreover, analog computers, hybrid analog digital computers, and, then, microprocessor-based systems were utilized for time domain analysis in epilepsy (Ktonas and Smith, 1974; Principe and Smith, 1982) and sleep staging (Smith and Principe, 1988). They were basically built by analog bandpass filters, followed by zero-crossing detectors, amplitude measurements and wave counts per unit of time for pattern matching. These methods are the precursors to the method proposed in this paper. They were online (and even compressed time) but required engineering teams to build and operate special purpose computers. The spectral approach started earlier (Grass and Gibbs, 1938) and was solidified in Walter (1963). It became widespread with the advent of the Fast Fourier Transform (FFT) algorithm and minicomputers to estimate in real time the power spectral density (PSD) of the EEP over windows. In the third author's opinion, this simplicity overshadowed their limitations and led to slow progress in computer-based EEG analysis—for more details see Principe and Brockmeier (2015).

Indeed, the PSD-based methods hides a major shortcoming: the time-frequency uncertainty relation proved by Gabor (1946), which states that the product of the time and frequency resolutions is lower bounded by a constant, i.e., one has to trade-off time resolution by frequency resolution. Since the EEPs are non stationary, the window size has to be sufficiently short to capture the stationary segments at the expense of poor frequency resolution (i.e., the ability to differentiate rhythms that are close together in frequency). The Time-Frequency (TF) decompositions provide the best compromise (Cohen, 1995), while the wavelets improve slightly on this limit (Unser and Aldroubi, 1996; Mallat, 1999). However, both methods still have a major problem: the time resolution of an event quantified by a peak in the PSD is given by the window length because the location in time that contributes to the power at a particular frequency is unknown, i.e., the phase of the PSD is constant over such window. Therefore, the PSD analysis is a bottleneck to provide sufficient time resolution to analyze the EEP in the cognitive brain; a potential alternative, though very inefficient computationally, would be window staggering. One could exploit the Hilbert transform (Bracewell, 1986) to improve on the Gabor uncertainty, but the problem of phase unwrapping and further interpretation is problematic, even presently.

This paper introduces an EEP model that not only incorporates neurophysiological principles, but also achieves exceptional temporal resolution limited by the sampling period alone (a direct consequence of its transient model-based approach). The relevant phasic events in the EEP are modeled by a set of temporal filters and an additive noise term using a training set obtained by sufficiently long single-channel records of EEPs from the desired conditions. Once the filters are obtained and the noise is characterized, the model can be applied to a test set producing as output a set of delta functions from each one of the filters, giving rise to a Marked Point Process (MPP) description for neuromodulation timing and amplitude organized by filter type. This point process interpretation resembles the work done by Tagliazucchi et al. (2012) and Wu et al. (2013) that applied deconvolution techniques to BOLD time series in fMRI data to estimate order parameters and functional connectivity in resting states of the brain, respectively. We devised robust techniques to effectively discriminate and separate in an unsupervised manner the background noise from the phasic event component that, ideally, contains only event-related oscillations for a particular frequency band. Furthermore, the modeling step requires only two free hyper-parameters (to be set by the users) and provides quantification of EEPs based on amplitude of phasic events and their timing per bandpass filter. In this way, we propose a model that goes beyond the conventional EEP spectral power decomposition techniques and integrates neurophysiological principles into a robust unsupervised learning method. This approach is relevant because the MPP description of the EEP serves as a mesoscopic representation of brain activity that can be easily combined with microarray single-spike recordings with which it shares the point process nature; this facilitates a multiscale representation of brain activity. Moreover, the fine temporal resolution of the MPP is also crucial to relate brain activity with cognitive events measured in the external world.

In order to test the model quality, we select two well-known EEP applications: single-channel EEG sleep spindle detection and multi-channel ECoG gamma-related motor correlates; both datasets have been throughly analyzed in the literature using power spectrum techniques and serve as model validation. These tests also illustrate the potential of the MPP analysis of EEPs, which provide information similar to PSD but can also address questions that normally require much more complex and invasive setups (such as propagation delays and conditioning of EEP events in spike firings). The rest of the paper continues as follows: section 2 explains the novel model framework for EEP and the corresponding methods. Section 3 details the two applications and the main results, while section 4 concludes the paper by summarizing the main ideas, discussing potential drawbacks, and proposing future work.

## 2. Methods

### 2.1. Transient model for EEP

As we have discussed, the concept of phasic events is well established in Clinical Neurology as well as in Computational Neuroscience. Therefore, our goal is to develop a generative model for a single EEP channel that captures the concept of transient (phasic) events so useful in brain science. Generative models can be implemented using statistical inference to learn the joint distribution of data and labels (Bishop and Lasserre, 2006) or using information theory principles to minimize redundancy (Barlow, 1961). The latter approach is very appropriate for this task because the goal is to explain the essential features of the input data without requiring labels and by exploiting the non-stationary dynamics of the underlying sources; a noteworthy example of such architectures is the well-known Independent Component Analysis (ICA). They were first applied to Computational Neuroscience by Olshausen and Fields in vision (Olshausen and Field, 1996), then Lewicki proved similar concepts for audition (Lewicki, 2002), and they have also been appropriate in the quantification of the sympathetic and parasympathetic contributions to the heart rate variability (Lucena et al., 2011). Brockmeier and Principe were the first to apply this general idea to EEP analysis (Brockmeier and Príncipe, 2016), and the present paper is the first description of the model for the general audience of brain scientists. It also improves on the practicality of the early work by simplifying the training procedure and including a noise model, which facilitates quantifying the ever present spontaneous EEP activity.

**Figure 1:**
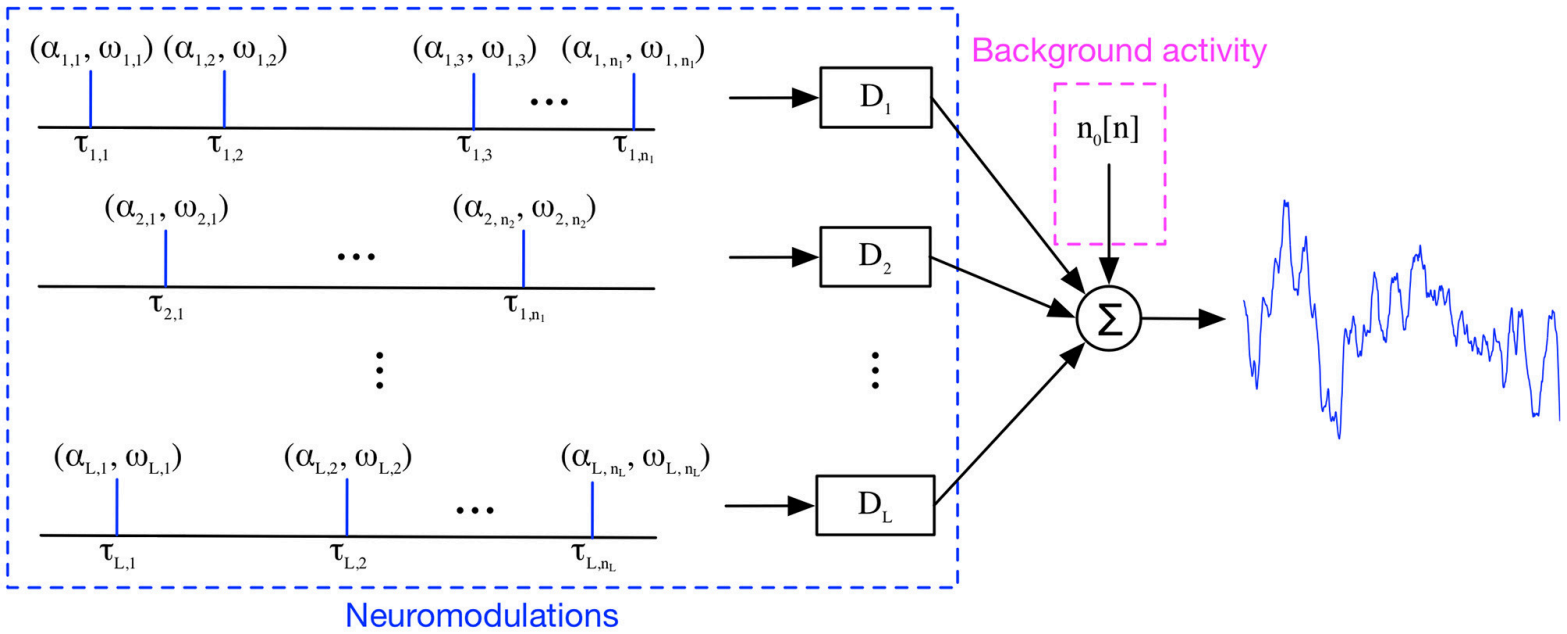
Transient Model for EEP. A single-channel, single-trial EEP trace is modeled as the noisy addition of reoccurring, transient patterns over time in a frequency-band specific approach.

Previous applications of generative models have been restricted to the solution of a set of coefficients for a linear or nonlinear model, without imposing architectural constraints to codify the prior knowledge of the phenomenon under analysis. Our approach is different because we want to obtain an analysis procedure that mimics the neurophysiology knowledge of phasic events that occur into specific frequency bands, superimposed on a featureless spontaneous activity background with a 1/*f* spectrum (Freeman, 1975). Another important assumption is based on the linearity of electromagnetic wave propagation in cortical tissue (Niedermeyer and da Silva, 2005), and the fact that the phasic events are sparse and reoccurring throughout the EEPs. All these assumptions are used in our definition of the model architecture that constitutes a generative model for EEPs (Figure 1). The collected and digitally sampled EEP data, *x*[*n*], is modeled as a sum of a noise term, *n*_0_[*n*], representing the spontaneous EEP activity with the outputs of a set of linear models, *y*_*i*_[*n*], i.e.,

(1) $$\begin{array}{rc} x\left[n\right] & =n_{0}\left[n\right]+\hat{x}\left[n\right]=n_{0}\left[n\right]+\overset{L}{\underset{i=1}{\sum }}y_{i}\left[n\right] \end{array}$$

(2) $$\begin{array}{rc} y_{i}\left[n\right] & =\overset{n_{i}}{\underset{j=1}{\sum }}\overset{\infty }{\underset{m=-\infty }{\sum }}\alpha_{i,j}\delta \left[n-\tau_{i,j}-m\right]d_{i,\omega_{j}}\left[m\right] \end{array}$$

where *y*_*i*_[*n*] constitute the frequency-specific temporal contributions of weighted Dirac delta functions convolved with an indexed family of Finite Impulse Response (FIR) linear bandpass filters, {*d*_*i*,_ω__*j*__}, i.e., a filter bank which constitutes a dictionary (*D*_*i*_ = {*d*_*i*,_ω__*j*__}) of time domain signatures representing the neuromodulations under analysis. Each dictionary is naturally assigned to one of the *L* frequency bands or rhythms under study for EEP modeling, i.e., delta, theta, alpha, sigma, beta, gamma bands, and ripples to simplify parameter learning. Each EEP rhythm band is further represented by *K* different bandpass filters with diverse central frequencies and/or different lengths; this constitutes one of the hyper-parameter of the model. Each dictionary entry includes another hyper parameter, *M*, corresponding to the duration of the transient events (i.e., the FIR filter order) in number of samples. Unlike previous attempts to use dictionary learning in EEG analysis (Durka and Blinowska, 1995) where the temporal signatures were handpicked from fixed basis (wavelets), here the FIR filter coefficients will be learned directly from data in a training phase. This Multiple Input Single Output (MISO) model resembles Freeman's wave packets ideas regarding the organization of EEG (Freeman and Rogers, 2002), among others. An important component of our model is the explicit modeling of the additive noise component, *n*_*o*_[*n*], that is characterized by the EEP's statistical properties. The MISO model assumes that there are a set of delta functions produced by the cognitive brain that excite the filter bank at certain times and with a given amplitude. As we demonstrate below, we do not need this specific information during model training, it is only necessary that each filter be sufficiently and sparsely excited in the EEP data used for training.

**Figure 2:**
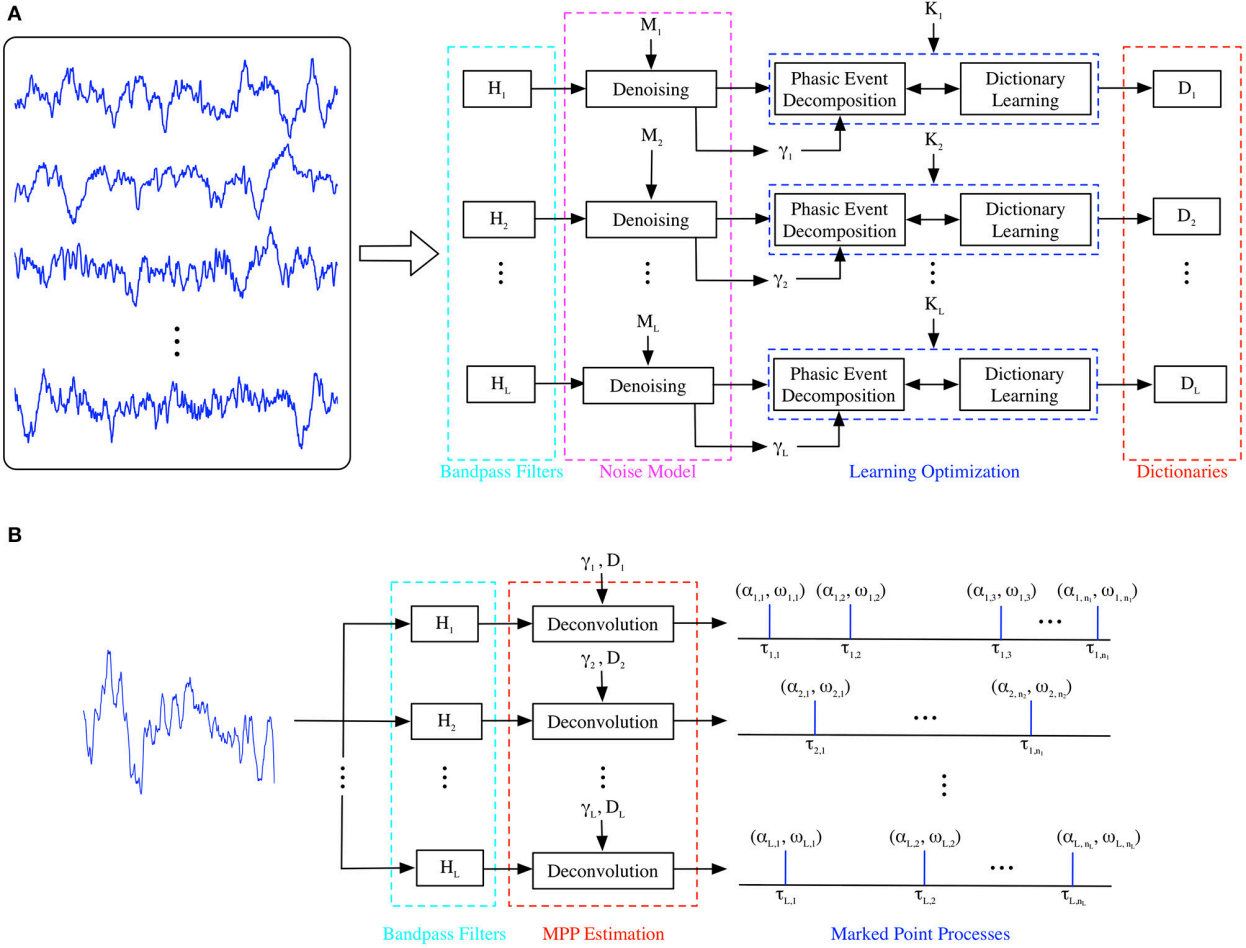
**(A)** Training of Generative Model. A set of single-channel EEP traces corresponding to a particular condition is denoised and its dictionary atoms are estimated in a frequency band-specific approach. **(B)** Test of Generative Model. A single-channel, single-trial recording is represented as sparse MPPs for each oscillatory rhythm under consideration.

Once the generative model is trained (Figure 2A), it can be applied to analyze a single channel EEP as shown in Figure 2B; this architecture will be called here the test model. If multiple channels are available, the same test model can be replicated for each available channel, providing a spatial temporal quantification of the brain in cognitive tasks, using EEPs. Functionally, the test model is simply the dual of the MISO model presented in Figure 1. The incoming EEP signal is first denoised by exploiting the noise model and then presented to a set of the bandpass filters to estimate the occurrence of neuromodulation; the output is a set of events in time that represent the time, amplitude and dictionary-related index of the phasic events that appear in the EEP channel under analysis. We select the occurrence of the events as the timing of the largest peak of the neuromodulation in each activated filter using an amplitude threshold; this creates, for each EEP channel, an event-dependent spike train that carries amplitude and frequency-specific information, unlike current spike train models of neural activity. The temporal resolution of the occurrence of the spikes in the test model is given by the sampling period selected by the digitization of the EEP, which is a great asset of the proposed technique, e.g., for 500 Hz, one can determine the occurrence of a phasic event (neuromodulation spindle) with 2 ms. resolution. This is similar to the resolution of the early SAMICOS for sleep analysis (Principe and Smith, 1986) but with a much improved statistical modeling approach of the EEPs. Therefore, the test model output can be further analyzed by exploiting the mathematical concept of Point Processes (Daley and Vere-Jones, 2007); specifically, the Dirac deltas can be regarded as a temporal Marked Point Process (MPP) with timings τ_*i*_ and at least bivariate attributes of amplitude and center frequency from the filter bank (α_*i*_, *f*_*i*_).

Finally, we would like to address in more detail the differences between the current MISO model and the early work of Brockmeier and Príncipe (2016) in order to provide context and alternatives for the difficulties involved in this approach. Conceptually, generative models attempt to represent an input signal *x*[*n*] by an internal, linear or nonlinear, static or dynamic model with output *y*[*n*] = *f*(*Ax*[*n*]), such that the reconstruction error is as small as possible, i.e., |*x*[*n*] − *y*[*n*]| ≈ 0, according to some loss function. Essentially, one attempts to learn a set of basis functions that span the input space, and the “dream” is to create a set of orthogonal basis in a functional space, which cover the most volume in solution space for a given dimension. This approach short-circuits the need to find a priori a set of hand-picked features that, albeit convenient for model training, always lose information and have been a source for non-optimal performance in EEG data analysis for many years. The generative learning approach is computational expensive in the training because the optimization needs to construct the essential representation for the incoming data using an unsupervised framework (the input is indeed the target response of the learned model). In order to facilitate the learning of essential features, one has to introduce a penalty in the optimization such that the columns of *A*, which can be thought of as the basis of the representation space, are sparse and generalize well. Moreover, one should also consider that the learning system architecture, i.e., the mapper that defines *f*(.), can also help define proper solutions for the learning problem by incorporating prior knowledge about the domain. Our MISO generative model is a good example of this, where we constrained the mapper to be a simple linear dynamical system—the FIR filter bank. Moreover, a mapper tuned to the application simplifies the user's selection of hyper parameters, which inevitably arises to tune the model performance, i.e., they tend to be related to neurophysiology and the specific goals of the analysis.

There are two basic family of approaches to learn the dictionary elements, also known as atoms: dictionary learning also called sparse coding (Elad, 2010), and Independent Component Analysis (ICA). The first approach assumes a generative model for the input with additional sparsity constraints of the sources. The optimal solution complies with such priors while minimizing the least-squares reconstruction error. Previous studies have shown the plausibility of efficiently estimating descriptors in the form filters in a data-driven approach (Lewicki, 2002; Smith and Lewicki, 2006; Balcan and Lewicki, 2009; Ekanadham et al., 2011). In particular, Matching Pursuit (MP) (Mallat and Zhang, 1993) has been proved useful in learning overcomplete basis in a greedy scheme (Aharon et al., 2006) and has even been extended to the time-series case (Mailhé et al., 2008). In these cases, the sources must be explicitly estimated. The second approach avoids this explicit estimation and exploits the statistical properties of the EEP. Basically, the filters are estimated by exploiting a matrix-based projection pursuit algorithm, where windowed snippets from the EEP are regarded as vectors in ICA. Hence, the learning takes place without explicit source estimation nor appealing for reconstruction cost functions. Other studies (Bell and Sejnowski, 1996; Davies and James, 2007) and our own (Lucena et al., 2011) have showcased the potential of efficiently estimating the basis exploiting the FastICA implementation (Hyvarinen, 1999). A comparison of the two family of approaches using synthetic and real EEPs can be found in Brockmeier and Príncipe (2016); this work shows that there are computational advantages to the first method, while performance was only slightly degraded. However, we can simplify the first approach even further and reduce the computational complexity as well as the number of free parameters by taking advantage of the architectural constraints and the neurophysiology. First, the generators of neuromodulation can be naturally organized by frequency bands or rhythms, i.e., the same cell assembly will not produce at the same time neuromodulations in two different frequency bands; therefore, the parameter estimation can be framed as an *L* independent Single Input Single Output (SISO) model optimizations instead of the more complex MISO case (Loza and Principe, 2017). Neuromodulations in EEPs are temporally sparse, i.e., they rarely overlap, so they can be represented by a single weighted *M*-sample-long FIR filter; this last condition resembles the sparse decomposition assumptions of MP. Finally, we can impose priors on the estimated filter bank parameters, *K* and *M*. By visually analyzing the length of the neuromodulation under analysis, we can properly set *M*, e.g., sleep spindles usually last at least 500 ms, so the filter order should be ≈250 for 500 Hz sampling. On the other hand, the number of subfilters in each band (*K*) should cover well the bandwidth of interest given the application (for modeling, more filters should be used compared with detection). Next, we detail the noise model and unsupervised learning methods exploited to accomplish the aforementioned estimation tasks.

### 2.2. Noise model

One of the most remarkable properties of spatio-temporal brain dynamics involves the constant shifts between highly complex unpredictable temporal chaos and a more robust, transiently predictable, and oscillatory stage (Buzsaki, 2006). This state of transitions is a special form of stability—criticality, which enables inevitable reorganization of the brain dynamics due to either external perturbations or internal processes; regardless of the source, the final result is revealed as large synchronized events, e.g., oscillations. Recent work (Luczak et al., 2009) has shown that the background activity is spatially stable at a longer time scale and may also be used as a signature for cognitive states. However, the temporally disorganized spontaneous activity effectively works as “noise” for the phasic events detection algorithms. In the context of the goals of this paper, we can discriminate two main components in the EEP: the background noise or ongoing activity, and the oscillatory component, which represents the synchronization of neuronal pools across space and time. With this neurophysiological concept in mind, we propose a decomposition for a particular bandpassed single-channel, single-trial trace, ỹ[*n*]:

(3) $$\begin{array}{r} ỹ\left[n\right]=y\left[n\right]+z\left[n\right] \end{array}$$

where *y*[*n*] is an ideal, noiseless contribution that only contains neuromodulations (a phasic event component), while *z*[*n*] represents a bandpass version of the noise component, *n*_0_[*n*], in Equation (1) for the EEP rhythm under analysis. During testing, the goal is to subtract *z*[*n*] before decomposing the EEP time series. We exploit the statistical properties of *z*[*n*] in order to separate both components in an unsupervised scheme following Freeman suggestion that there is a strong correlation between EEP Gaussianity and resting behavioral states (Freeman and Quiroga, 2012). When the brain is actively processing information, there are deviations from Gaussianity, which we hypothesize are the neuromodulations that we strive to model. The challenge is to find a methodology to estimate a threshold that can quantify Gaussianity in EEPs independently of the high variability of the neuromodulation and still discriminate accurately between background and phasic event components. As suggested by Freeman (Freeman and Quiroga, 2012), our approach calls for higher-order statistical moments (kurtosis), however, instead of being applied to individual time samples, we propose its application to snippets of embedding vectors of size *M* (*M*-snippet), where *M*, in samples, is the selected filter length.

We begin by embedding the training signals in *M* dimensions, the norm is computed for each vector, and, then, the statistical test is applied. The embedding starts by extracting the obvious *M*-sample-long modulated patterns first (neuromodulation) through their instantaneous amplitude using the Hilbert transform (Bracewell, 1986); then, the remaining unmodulated samples are embedded in the *M* dimension as well. The norm is computed for all the *M*-dimensional vectors and their density is estimated, e.g., histograms or Kernel methods can be exploited here. The distribution of these norms will resemble a skewed density with the main lobe being close to Gaussian and the long tail representing high-norm events. Lastly, the statistical test exploits kurtosis to estimate the limit between the Gaussian mode and the tail of the distribution. This test, however, is strong only for a sufficiently large number of samples for *M* in order to guarantee the resulting chi-square distribution (norms density) resembles a Gaussian according to the Central Limit Theorem; fortunately, this condition is not a limitation in our time-based approach, where sampling frequencies and *M* values are inherently large. The final result is a threshold parameter for each rhythm under study, γ_*i*_ (Figure 2A), that plays an important role on the overall methodology because it segments the training data in regions that, with high likelihood, contain neuromodulations; this, consequently, will improve parameter estimation. Therefore, each of the *M*-snippet samples with norms below γ_*i*_ are removed from the training set before starting parameter estimation, i.e., the EEP traces now display discontinuities due to the choice of the embedding parameter. The remaining *M*-snippets are bona fide phasic events that will be used for training the model. It is worth noting that alternative approaches that preserve the smoothness of the input can be utilized as well, however, they are more computationally demanding; for further references, see Loza (2017).

### 2.3. Unsupervised learning of model parameters

As it was previously mentioned, our learning scheme takes the form of dictionary learning or sparse coding approaches, i.e., the sources (MPP) are explicitly estimated during the estimation of the dictionary elements. Therefore, the goal of this step is to estimate the *K M*-sample-long FIR filter parameters for each oscillatory rhythm under study. There are two very distinctive iterative stages: phasic event decomposition and dictionary learning. The estimation begins with an initial filter bank, whose parameters are obtained by a hand picked selection of *M*-snippets recognized by the researcher as neuromodulation from a bandpassed EEP corresponding to a particular channel, condition and rhythm in the training set. These *M*-snippets become the impulse responses of each of the *K* FIR filters in the filter bank. To simplify the selection procedure, we recommend the use of the Hilbert transform to isolate such modulated patterns and provide the initial filter bank. Alternatively, a dictionary from previous experiments can also be utilized. As a result, this seed dictionary contains *K* FIR filters with relatively large-norm impulse responses (90-th percentile) and clear modulation patterns.

#### 2.3.1. Phasic event decomposition

The adaptive unsupervised learning stage for the transient model is inspired by a shift-invariant implementation of k-means, the well-known clustering technique (Gersho and Gray, 1991). For each rhythm, the seed dictionary, with its *K* initial dictionary elements become centers in k-means. The stage known in k-means as cluster assignment is denoted as phasic event decomposition for our learning approach (Figure 2A). Using a procedure very similar to the greedy decomposition technique of Matching Pursuit (Mallat and Zhang, 1993), the remaining *M*-snippets in the training set are assigned to the closest center. We use the Fast Fourier Transform (FFT) to efficiently assign each of the *M*-snippets to the closest dictionary atom. Unlike previous attempts that take advantage of greedy sparse decomposition techniques (Durka and Blinowska, 1995), our proposed phasic event assignment is much simpler (and, hence, faster) because of the denoising step described above that explicitly discriminates between neuromodulations and noisy background activity. This novel methodology eliminates one of the free parameters of MP when it is utilized in sparse approximation problems—the sparsity of the decomposition; for our case, this value is always equal to one, i.e., there is no over-representation of events.

It is worth noting that this phasic event decomposition is the only step required in the testing phase; however, to avoid potential confusion, we will denote the phasic event decomposition algorithm as deconvolution in the testing implementation (Figure 2B). The projection of a particular *M*-snippet onto the closest dictionary atom in the test set (α or Euclidean distance to closest center) is compared to γ_*i*_; if α falls below the threshold, the *M*-snippet is not considered a phasic event; if it meets the criterion, then, the snippet is considered a MPP event. Therefore, its amplitude (α), index of the closest dictionary atom (ω), and timing (τ) are noted. Specifically, they provide the necessary information to create the marked point process as the output of the model.

#### 2.3.2. Dictionary learning

The second stage exploits the elements of each cluster to update the filter parameters with the signatures of all the similar *M*-snippets via robust low-rank transformations (Loza and Principe, 2017). The *M*-snippets from the previous stage are grouped in matrices according to their indexes, ω. Then, we apply scale-invariant low-rank transformations to each matrix associated with a dictionary element (center); in particular, Singular Value Decomposition (SVD) provides a low-rank transformation where the first component maximizes the represented variance or power in the set; this first component would constitute the updated dictionary atom. However, regular SVD optimizes only second-order statistics and is prone to outlier effects. In order to provide robustness to the framework, we utilize correntropy as the cost function of the low-rank transform (Liu et al., 2007). Correntropy is defined as a dependence measure for random variables and has been throughly applied in non-linear analysis, robust decomposition, and sparse approximation (Gunduz and Principe, 2009; He et al., 2011; Loza and Principe, 2016a). The robust correntropy-based SVD technique utilizes the Gaussian kernel in order to go beyond the benchmark of second-order statistical moments. The addition of correntropy to the framework does not add significant computational complexity nor it requires a validation stage when it comes to the free parameter introduced by the Gaussian kernel, σ, the kernel width. In fact, previous studies have taken advantage of the Silverman's rule (Silverman, 1986) and have been successful in mimicking the effect of kernel annealing and providing robustness against outliers (He et al., 2011; Loza and Principe, 2016b). In this way, a robust dictionary learning technique ensures that the updated FIR filters are not biased by outlier *M*-snippet shapes while preserving the number of free parameters in the overall model.

Both stages run in an iterative manner until a particular terminal criterion has been reached, e.g., minimum variation of dictionary atoms (e.g., Frobenius norm of successive parameter estimations less than 10^−2^) or fixed number of iterations (in practice, at least 20 for each initial condition has proven effective). It is worth mentioning that, due to the greedy nature of both stages, it is necessary to avoid suboptimal, local solutions by running the iterative optimization with different initial conditions. In the end, the set of atoms with the lower mutual coherence (Donoho and Huo, 2001) will be chosen as the optimal dictionary.

## 3. Results

### 3.1. Sleep spindles detection

The first validation experiment comes from one of the most distinctive EEG patterns of stage 2 non-REM sleep in humans—sleep spindles. They have been associated to memory processes (Schabus et al., 2004), cortical development (Khazipov et al., 2004), and have been regarded as potential biomarkers of psychiatric disorders (Ferrarelli et al., 2010). However, most of the detectors used in these modern studies (see Principe and Smith, 1986 for an older review) rely on cross-validation schemes to set a proper detection threshold, e.g., via the receiver operating characteristics (ROC) curve. On the other hand, our approach does not require previous enumerated data for free parameter cross-validation, it rather utilizes neurophysiological principles to estimate and model the precise temporal markers of sleep spindles in single-channel EEG traces.

The data under study belongs to the TCTS Laboratory of the University of MONS and the Sleep Laboratory in the Université Libre de Bruxelles. The publicly available DREAMS database (Devuyst, 2011) was utilized to validate the proposed methodology via quantitative comparison to sleep scoring annotations. Furthermore, the traces were recorded using a digital 32-channel polygraph (BrainnetTM System of MEDATEC, Brussels, Belgium) that includes two Electrooculogram (EOG) channels (P8-A1, P18-A1), three EEG channels (CZ-A1 or C3-A1, FP1-A1, and O1-A1) and one submental Electromyography (EMG) channel. The records were anonymized before being saved in the standard European Data Format (EDF). The database provides one 30-min single-channel EEG segment from whole-night recordings for each one of the 8 patients in a sleep pathology study (different bipolar electrodes per subject). The sampling frequencies range from 50 to 200 Hz, and, in an effort to reflect reality as close as possible, these traces are not noise-free, pre-processed, nor manually chosen. Lastly, two expert clinicians independently score the 30-min single-channel traces and provide annotations in the form of timestamps and durations of sleep spindles. It is worth mentioning that only the 6 patients with both scores available are part of the present analysis.

In order to have proper comparisons to the ground truth, the *M* parameter was selected as the median value of the durations for each visual scorer per subject. This value ranged from 700 to 1,010 ms for the first expert, and it was equal to 1,000 ms for the second scorer. *K* was chosen as a multiple of *M* for each patient, i.e., *K* = ⌊*K*′ × *M*⌋, where *M* is in samples and *K*′ takes values from 0.125 to 1 with 0.125 intervals. In order to individualize the sigma band, the only one interesting in this study, the single-channel, single-trial EEG traces were pre-processed via bandpass filtering in the sigma band (11–16 Hz) utilizing a Butterworth filter with a designed quality factor close to 2. Lastly, a total number of 20 different initial conditions were given to the iterative optimization scheme for each combination of *M*, *K*, subject, and visual scorer.

**Figure 3:**
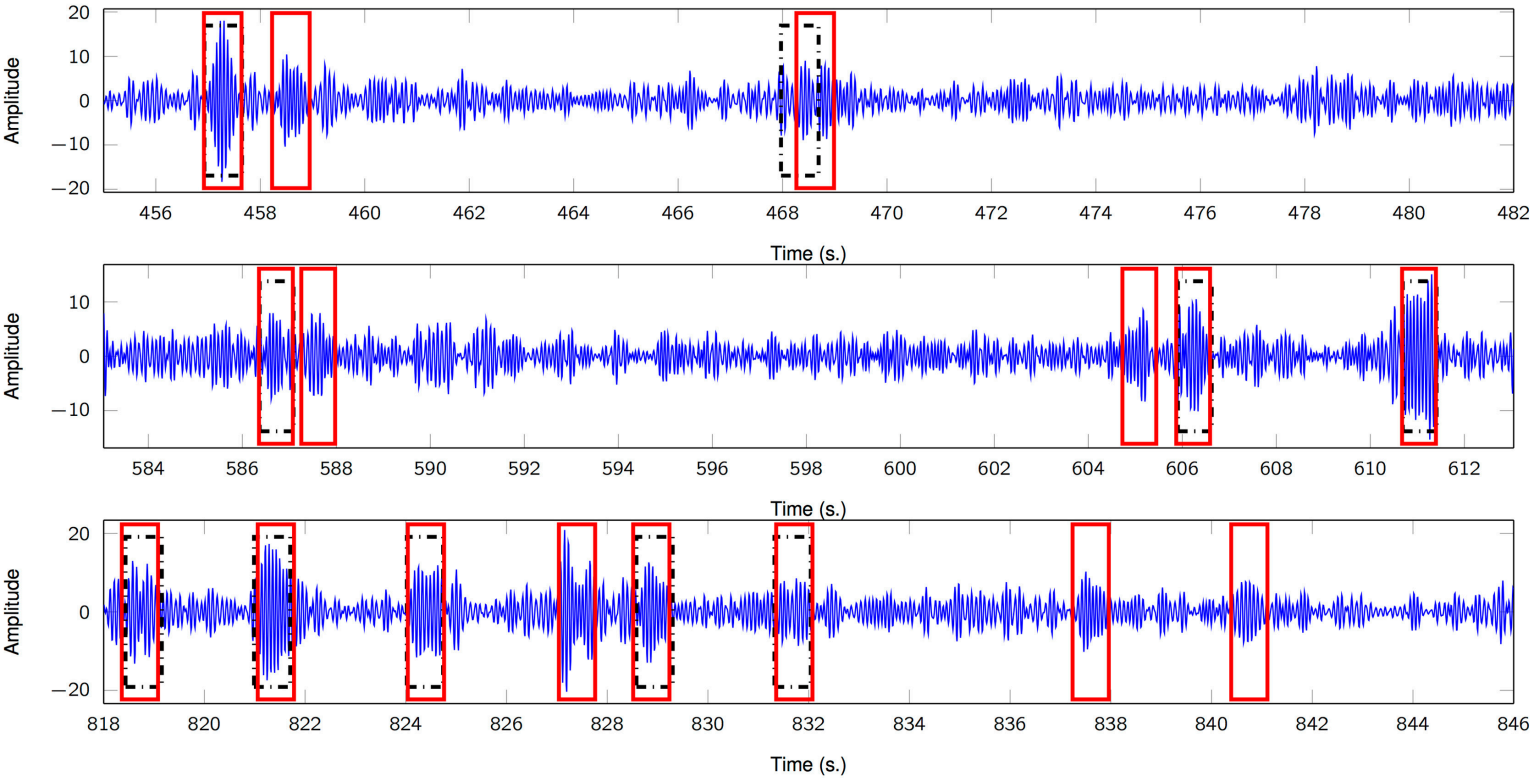
Comparison of sleep spindle unsupervised detector and expert 1 (DREAMS database). Three 30-s segments show putative sleep spindles scored by the clinician (black rectangles) and by the proposed unsupervised scheme (red rectangles). Subject 5.

**Figure 4:**
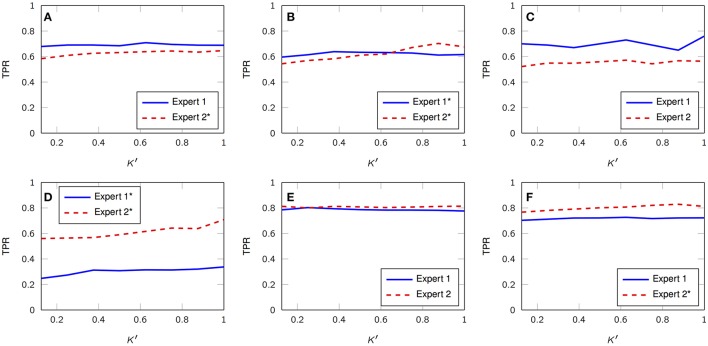
Average TPR vs. *K*′ with respect to both visual scorers (DREAMS database). **(A–F)** Subject 1, 2, 3, 4, 5, 6. Legends with black asterisk denote statistical dependency on *K*′. (1-way ANOVA, *p* < 0.01).

Figure 3 depicts three representative 30-s segments from one patient and its corresponding timestamps according to expert 1 alongside the estimated, unsupervised marks obtained from the proposed framework; in general, there is a consistent agreement between both criteria. We also analyze the True Positive Rate (TPR) according to both scorers and its dependence on *K*′, i.e., the number of estimated FIR filters. Figure 4 summarizes the results and depicts the cases where statistical association was found (1-way ANOVA *p* < 0.01). The sensitivity peaks at different values of *K*′ for each case and reveals an unclear consensus whether *K*′, and therefore *K*, plays a major role for this specific measure. The particular performance of subject 4 is discussed in the final section of the paper.

Tables 1, 2 summarize the sensitivity and specificity of the unsupervised detectors with respect to each visual scorer. Specifically, the tables also evaluate the variation as a dependence on the number of estimated dictionary atoms, *K*′, i.e., *K*. The sensitivity achieves maximums of 0.72 and 0.69 in comparison to experts 1 and 2, respectively (median over subjects), which is comparable to the benchmark of inter expert agreement reported by the database authors (Devuyst et al., 2011)—0.7 TPR. On the other hand, the specificity of our approach reaches 0.85 and 0.88 with respect to visual scorers 1 and 2, respectively, which falls below the 0.98-mark reported by the database authors.

**Table 1:** Sensitivity of automatic sleep spindle detector with respect to both visual scorers for different number of dictionary atoms (DREAMS dataset).

***K*′**								
**Expert**	**0.125**	**0.25**	**0.375**	**0.5**	**0.625**	**0.75**	**0.875**	**1**
1	0.69	0.69	0.68	0.69	0.72	0.69	0.67	0.71
2	0.57	0.59	0.60	0.62	0.63	0.66	0.67	0.69

*Median over subjects*.

**Table 2:** Specificity of automatic sleep spindle detector with respect to both visual scorers for different number of dictionary atoms (DREAMS dataset).

***K*′**								
**Expert**	**0.125**	**0.25**	**0.375**	**0.5**	**0.625**	**0.75**	**0.875**	**1**
1	0.85	0.85	0.84	0.84	0.84	0.83	0.83	0.83
2	0.88	0.87	0.86	0.86	0.86	0.86	0.86	0.86

*Median over subjects*.

### 3.2. Motor correlates from ECoG

The second experiment corresponds to the Brain-Computer Interface (BCI) Competition IV, dataset 4 (Blankertz, 2009; Tangermann et al., 2012). In particular, three epileptic patients at the Harborview Hospital in Seattle, Washington were implanted with subdural electrode grids on the surface of the brain with the main purpose of clinical motoring and localization of seizure foci (different number of channels per subject). The study was approved by the internal review board of Harborview Hospital; in addition, the patient data was anonymized according to internal review board protocols to comply with HIPAA regulations. The recordings were amplified and digitized using Synamps2 amplifiers (Neuroscan, El Paso, TX, USA). The BCI system BCI2000 (Schalk et al., 2004) was utilized to provide the visual stimuli, acquire the brain signals, and record the flexion of individual fingers using a data glove (Fifth Dimension Technologies, Irvine, CA, USA). The patients were asked to move a particular finger after visual cues in a computer monitor in front of them. Each 2-s-long cue was followed by a 2-s-long rest period. During each finger flexion task, the subjects typically performed 3–5 consecutive movements or taps. Particularly, the goal of the challenge was to infer the finger flexion based on the multi-channel ECoG traces alone corresponding to the contralateral hemisphere of finger movement. Hence, timestamps of cued visual stimuli, finger flexion kinematics, and digitized (sampling frequency 1,000 Hz), bandpassed (0.15–200 Hz) ECoG traces were provided in the dataset.

Even though the so-called Local Motor Potentials (LMP) are discriminant when it comes to finger movement and other motor tasks (Schalk et al., 2007; Kubanek et al., 2009), we decided to focus on the high-frequency content of the ECoG recordings due to their inherent short-lived, transient events that can be properly modeled exploiting the proposed framework. Specifically, we focus on the high-gamma band (76–100 Hz) that provides more localized activity thanks to the short-lived synchronization windows this rhythm allows (Tangermann et al., 2012). Once again, a Butterworth filter with quality factor close to 2 was utilized after downsampling the original recordings to 500 Hz. Next, the two free parameters of the transient model are set: *M* is chosen equal to 50 samples, or 100 ms, after thorough visual inspection of the temporal traces and their corresponding TF analysis. *K* is set as discrete values from 2 to 100 in order to investigate the dependence of the metrics with respect to the number of estimated dictionary atoms. The methods are ran for each individual channel separately setting 5 different initial conditions and 20 iterations maximum.

**Figure 5:**
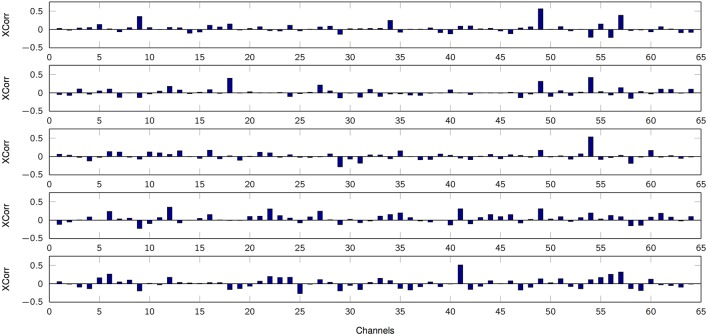
Average cross-correlation between smoothed high-gamma MPPs and finger flexion kinematics. From top to bottom: finger 1, 2, 3, 4, 5. *K* = 50. Subject 3.

The first set of results focuses on amplitude coding using the MPP properties, e.g., the density of spikes and their amplitudes. In the test, after the MPP is obtained, each delta function signifying an MPP is smoothed utilizing a truncated Gaussian kernel (σ = 100, finite support of 200 samples) in order to obtain comparable timescales between the Marked Point Processes and the finger kinematics. Effectively, we are estimating the MPP rate with this procedure. Then, the normalized cross-correlation between the smoothed MPP and the finger movement is computed for each trial paying special attention to causal results alone. Lastly, the average cross-correlation is obtained for each sensor-finger pair (mean over finger flexion tasks or trials). Figure 5 illustrates such relationship for one patient, and it is evident that the smoothed MPP is spatially sparse and localized. Moreover, similar trends are observed in the remaining subjects with the only significant difference being the spatial distribution due to specific electrode grid arrangements. Multiple 1-way ANOVA analyses for each value of *K* under consideration (7 cases: 2, 5, 12, 25, 50, 75, 100) and all possible finger-patient combinations (15 cases) reveal that 97% of the cases exhibit at least one channel per finger, patient and fixed *K* that does not conform to the equal mean hypothesis (*p* < 0.01). Hence robustly, at least one channel contains phasic events with timestamps that are positively correlated to a particular finger movement. Lastly, we determined the potential relationship between average cross-correlation (between smoothed MPP and finger flexion) and the number of estimated dictionary atoms. Specifically we focus on one channel per finger alone, i.e., each motor task is associated to the electrode with higher average cross-correlation; then, the statistical significance is computed (1-way ANOVA) with respect to *K*. As Table 3 indicates, there is not enough evidence to suggest a strong correlation between the cross-correlation measure and the cardinality of the filter bank.

**Table 3:** Statistical significance of the average cross-correlation between smoothed high-gamma MPPs and finger flexion kinematics with respect to *K*.

***K***								
**Finger**	**2**	**5**	**12**	**25**	**50**	**75**	**100**	***p*****-value**
1	0.57	0.58	0.61	0.62	0.63	0.61	0.61	0.95
2	0.47	0.51	0.52	0.51	0.52	0.55	0.53	0.99
3	0.39	0.45	0.48	0.49	0.47	0.48	0.49	0.51
4	0.59	0.62	0.62	0.65	0.66	0.64	0.64	0.95
5	0.44	0.44	0.45	0.45	0.47	0.46	0.46	0.91

*1-way ANOVA. Subject 2*.

One of the advantages of the exceptional temporal resolution and point process nature of the MPP is the possibility to compute the characteristic delay between brain activity and motor task execution. Specifically, the time lags from the previous cross-correlation analysis can be regarded as such estimated delays. Table 4 shows the average temporal lags for the channel with the higher normalized cross-correlation per finger for all the subjects under study. The delay falls in the range of approximately 7–8.5 ms.

**Table 4:** Estimated delay between high-gamma ECoG neuromodulations and finger flexion tasks.

**Subject**	**Estimated delay (ms)**
1	7.04
2	8.40
3	8.33

*Average over K and fingers*.

**Figure 6:**
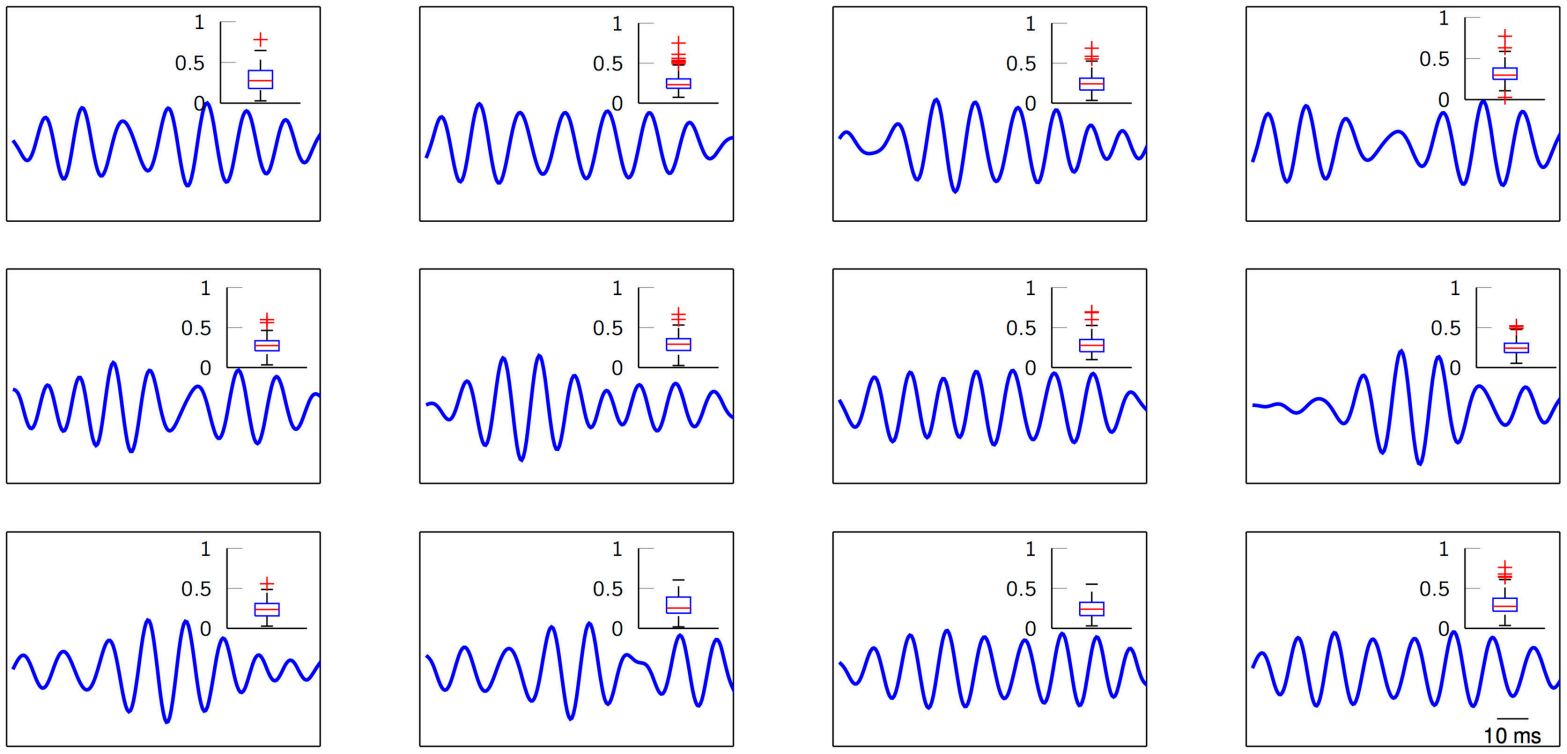
Estimated normalized high-gamma dictionary atoms from ECoG traces. Insets show the distribution (box plots) of the Euclidean distances between the *M*-snippets assigned to each FIR filter and their corresponding learned dictionary atom. Red crosses indicate potential outliers. Subject 3, electrode 49. *K* = 12 for visual purposes.

The next set of results pays close attention to the actual learned FIR filters. The estimation process can be easily biased by outlier shapes and noise-related phenomena that might distort the nature of the “ideal” induced potentials. For this reason, correntropy provides a sound safeguard and ensures robust estimation of the prototypical *M*-snippet templates. Figure 6 depicts the learned modulatory patterns with a distribution of their closest *M*-snippets in the form of box plots of Euclidean distances. These distances are computed considering the extracted *M*-snippets corresponding to each particular dictionary atom, i.e., a sort of cluster compactness measure. Not only the prototypical templates seem to exhibit different modulatory levels that can be further analyzed, but their distributions include clear outliers (red crosses) that might bias the estimated FIR filter. However, by exploiting correntropy as the cost function of the SVD method, we can guarantee that the resulting estimated shapes are, indeed, unbiased.

**Figure 7:**
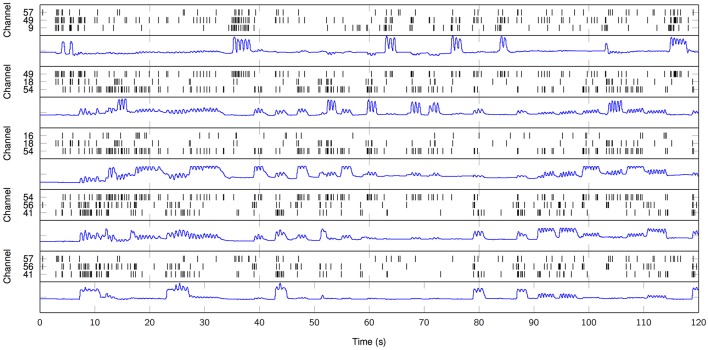
Raster plot of high-gamma MPP samples from ECoG traces (black) in comparison to finger flexion kinematics (blue). Three particular channels per finger. From top to bottom: finger 1, 2, 3, 4, 5. *K* = 100. Subject 3.

**Figure 8:**
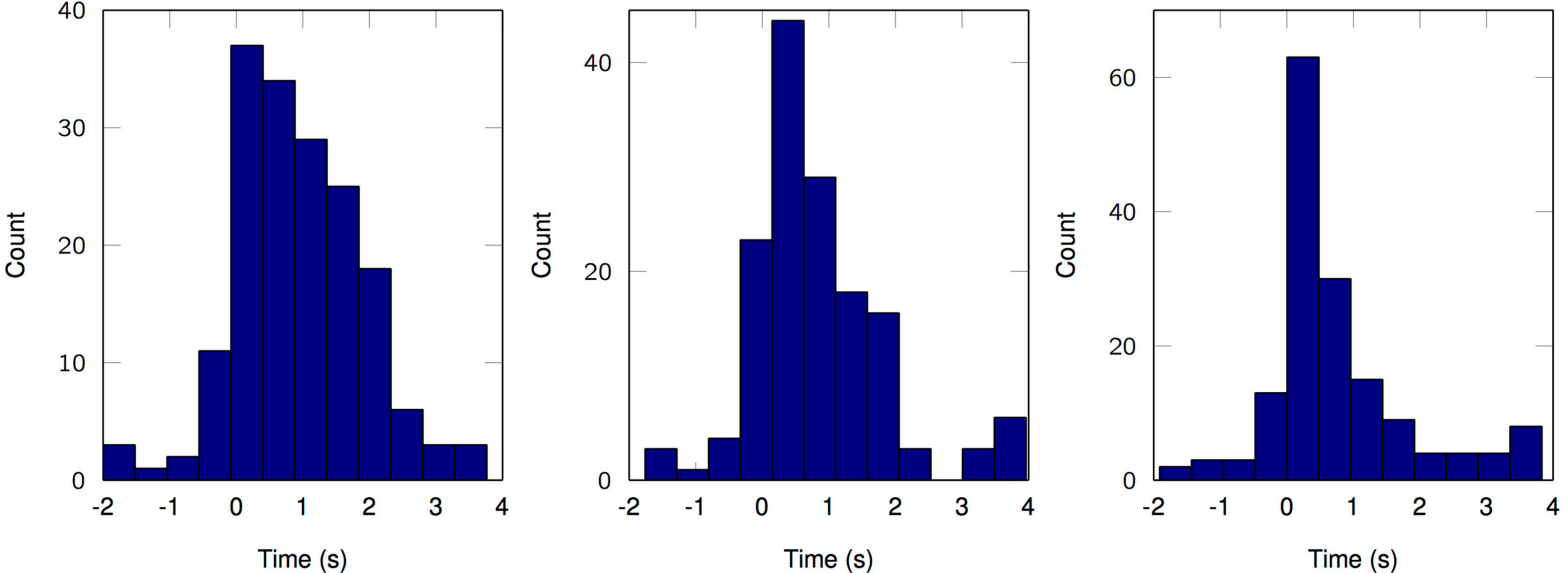
Estimated density of phasic event occurrence before, during and after motor task (2-s-long windows). **Left**, Subject 1, finger 1, channel 43. **Middle**, Subject 2, finger 3, channel 8. **Right**, Subject 3, finger 1, channel 9. *K* = 100.

Lastly, we explicitly exploit the sparse nature of the resulting MPP in order to elucidate a rate coding mechanism for motor control. According to the three channels with the higher average cross-correlation per finger from the first set of results, we plot the temporal distribution of MPP timings (τ) in comparison to the finger flexion kinematic traces for a 2-min period (Figure 7). It is evident that some channels remain “silent” during periods of motor inactivity and subsequently start to become active or *fire* when the motor activity starts; this representation (raster plot) and methods are very similar to the ones usually applied when working with spikes. With that in mind, it is possible to make use of other specific tools and techniques normally reserved for Point Processes (PP) only. For instance, it is possible to estimate the phasic event density (intensity function of the PP) before, during and after finger flexion movements. Figure 8 clearly resembles a Peristimulus Time Histogram (PSTH); particularly, the density significantly increases in the vicinity of the zero-mark, i.e., visual cue, and subsequently decreases after the motor task is finished approximately 2 s later.

## 4. Discussion

The methods and results of this paper suggest that a novel event-based interpretation of EEPs and neuronal oscillations is advantageous from a modeling point of view. The temporally sparse nature of the relevant neuromodulations fits perfectly in a transient model that strives to model a single-channel, single-trial EEP trace as the superposition of reoccurring, short-lived patterns in different frequency bands within a background noise environment. In particular, the Marked Point Process framework facilitates the quantification and further interpretation of the estimated phasic events. Two main applications are exploited in order to validate such novel point of view: EEG sleep spindle detection and ECoG motor correlates in the high-gamma band.

In general, there is agreement between timestamps from visual scorers and the proposed unsupervised approach in the DREAMS sleep spindles database (Figure 3); however, some snippets are categorized as sleep spindles due to their modulatory nature alone, when, in actuality according to the clinician, they are not putative spindles. This might be a result of particular *M*-snippets located at the fringe between the phasic event component and its noise counterpart in the distribution of the norms mentioned in section 2.2. Moreover, we would like to add that no minimum amplitude threshold nor minimum length constraint has been applied in our model, while the clinician may subconsciously be using some thresholding. Also, it is clear that the model underperforms with subject 4 in Figure 4; this is a direct consequence of the biased estimation of γ due to the excessive EMG artifacts (mentioned in the original manuscript from the database authors) that leaked to the sigma band and affected the norm distribution. We expect the metrics to improve if proper pre-processing takes place, e.g., EMG interference is reflected across the whole spectral support of the signal and, therefore, can be detected via broadband TF analysis or comparison of relative power over time and frequency.

There is a direct correlation between higher median sensitivities and larger dictionaries; however, the overall change is only a couple of percentage points, which might not justify the extra computational burden that comes with a larger number of filters in the filter bank. Conversely, the specificity seems to decrease with increasing dictionary sizes; nevertheless once again, the change is only a couple of percentage points. We believe the difference with the benchmark results from Devuyst et al. (2011) in terms of specificity is mainly due to a lack of pre-processing mechanisms to handle artifacts.

In summary, the proposed methods are capable of detecting and modeling sleep spindles in an automatic, unsupervised manner without appealing to add-hoc cross-validation schemes in the search for optimal thresholds. We utilize neurophysiological principles, such as the Gaussianity of the background noise, to select threshold parameters that separate putative sleep spindles from noise components. This highlights the flexibility of our methodology, where we did not modify the overall structure of the model nor the amount of free parameters. Instead, the transient model was utilized to achieve benchmark metrics and proved to mimic the clinical interpretation of electrophysiological biomarkers.

The results from the BCI Competition IV, dataset 4 showcase the innovation and potential of the MPP framework for relating brain activity with behavior. For instance, the MPP rate analysis agrees with a previous study where it was noted that adjacent fingers present common electrodes as a consequence of correlated, involuntary flexions (Miller and Schalk, 2008). Also, the statistical analysis for all the different values of *K* and all possible finger-patient combination suggests a robust trend of MPPs strongly correlated to the finger flexion kinematics. Similar conclusions have been achieved with different methods and processing techniques in the same dataset (Flamary and Rakotomamonjy, 2012; Liang and Bougrain, 2012; Tangermann et al., 2012); thus, confirming the validity of our overall phasic event framework. In terms of filter bank cardinality, the emerging trend seems to indicate that the average normalized cross-correlation improves slightly when a higher number of dictionary atoms are estimated (Table 3 and Supplementary Material, Tables S1, S2). However, practical limitations need to be taken into consideration; especially for this dataset where high sampling frequencies and multi-channel recordings are some of the main data features.

The estimated delay between brain activity and motor task falls in the 7–9 ms. range, which is consistent with previous experiments where the conduction time from cerebral cortex to forelimb muscle was assessed (Cheney and Fetz, 1980; McKiernan et al., 1998; Park et al., 2004). However, such studies exploit cell firing and other spike-related measures alongside EMG activity in order to estimate the aforementioned delay. In our case, we exploit the intrinsic exceptional temporal resolution of the transient model that can achieve a lower bound equal to the sampling period; this appealing property can not be obtained with regular Time-Frequency decompositions alone.

The ongoing plastic nature of the brain reflected in phasic event production, make the modeling task a necessity. For our case, this modeling comes in the form of the estimated FIR filters in a data-driven scheme by exploiting unsupervised learning techniques. Hence, it is crucial to estimate such patterns in a principled and robust scheme. Figure 6 makes a case for the justified use of correntropy as the cost function in the dictionary learning stage. In particular, the presence of outliers in the box plots are the result of a poor linear modeling of non-linear dynamics in the brain, i.e., spike-to-wave transform (Freeman, 1975), and phasic event distortion due to colored noise. However, correntropy ensures that the final estimation will be, ideally, unbiased by the outliers and closer to “ideal neuromodulations.” Therefore, this application highlights the need for robust processing when it comes to a signal characterized by noise and artifacts, such as EEP.

The rate coding results presented in Figure 8 are disruptive for the EEP field. The PSTH-like nature of the model output is only one example of future point process-based methods that can be applied directly to the estimated MPP, e.g., phasic event rate differences, tuning curves, inter phasic event interval densities with the classical parametric and non-parametric estimations (Daley and Vere-Jones, 2007). These methods can also be immediately integrated with spike train single neuron analysis opening the door for a multiscale modeling of electrical brain activity. For instance, the neuromodulation events captured by the proposed model can be used to parse out which neurons are involved in the mesoscopic events by statistical conditioning. In fact, because the neural action potentials and the phasic events are both represented mathematically by point processes, one can exploit techniques very similar to Spike Trigger Averages (STA) and Joint Peristimulus Time Histograms (JPSTH), but instead of using external stimuli, we can directly use the phasic event MPP. We expect that this will provide added multiscale information of the role of fields in neural organization. In short, we have introduced a novel interpretation to neuromodulations that would not be possible via classical decomposition techniques or Time-Frequency analysis. For the BCI applications, the proposed framework opens the door to innovative on-line detectors/predictors BCI systems where it is only required to estimate the density of event-related oscillations in order to obtain relevant motor decoding and topographical discrimination of active brain areas.

Potential caveats arise from the additional constraints of the methodology; namely, it is necessary to focus on a single oscillatory rhythm at a time, which is also preferred in the clinical and research fields (Niedermeyer and da Silva, 2005; Buzsaki, 2006). However, in our implementation, it is a matter of utmost importance to properly select the pre-processing bandpass filter for each neurophysiological band. For instance, instead of higher-order narrowband filters with large stop-band attenuation, which introduce artificial patterns, such as ringing artifacts (Freeman and Quiroga, 2012; Widmann and Schröger, 2012), as can be expected from high amplitude and long impulse responses, it is imperative to select filters with quality factors (ratio between central frequency and main filter lobe) close to 1 (Principe and Smith, 1982). Moreover, FIR filters should be favored in on-line applications due to their inherent linear phase; however, IIR filters with zero-phase implementations should suffice in most off-line studies, although they are more time consuming because they require two filtering operations in reverse direction. Another key aspect is the seed dictionary; although filters from previous studies can be exploited for the initialization of the dictionary, the most appealing feature of the overall framework is its data-driven approach. Hence, we must ensure that the initial filters are sufficiently different enough from each other to cover well the selected frequency band. This can be achieved by favoring seed dictionaries with low mutual coherence (Donoho and Huo, 2001), i.e., small linear correlation between pairs of filters. Another alternative could exploit the modulation of the *M*-snippets, e.g., select *K* filters with a large range of quality factors.

The proposed noise model exploits EEP deviations from Gaussianity as a mean to delineate the constant transitions in the cortex from the resting state to work. For our particular validation experiments, such “work” state is tightly related to well-known processes, generation mechanisms and/or external stimuli, e.g., sleep spindles are regulated by the mutual interaction between GABAergic reticular neurons and excitatory thalamic cells (Buzsáki and Draguhn, 2004). However, we are not claiming that such active state is the result of external processes or sensory stimuli alone; as the metaestable state of the cortex implies, the inevitable transitions from chaos to synchronization can be brought about via internal, even spontaneous, processes. In fact, previous studies hypothesize that, at the neuron level, task-evoked events might be constrained by the patterns displayed during spontaneous activity (Luczak et al., 2009). Therefore, the definition of the rest state of the proposed noise model has to be explicit in terms of experimental design or well-proven neurophysiological mechanisms. In any case, these specifications do not limit the use of the proposed method; indeed, it could be utilized to detect and model transient events in the spontaneous activity of EEPs.

The two main free parameters of the model, *M* and *K*, must be carefully selected depending on the context, i.e., a gray box type of approach. *M* is strictly tied to the oscillatory rhythm under study; as previously mentioned, visual inspection and classical TF analysis should come hand-in-hand with general neurophysiological concepts and previous studies in order to properly set the duration of putative induced potentials. On the other hand, *K* heavily depends on the quality of the recordings, e.g., cleaner traces as in the ECoG dataset require a smaller number of dictionary atoms, while noisy EEP recordings usually demand for larger dictionaries in order to accommodate larger fluctuations. Ideally, our attempt is to quantize a high-dimensional “wave” space where the *M*-sample-long phasic events are represented. This space can also be interpreted as a continuous state-space model of the generative process from action potentials to neuronal waves (Freeman, 1975). This condition alongside the plastic nature of the brain and its electrical potentials make the quantization task even more daunting. This might be the reason behind the increase in performance metrics when the dictionary size increases accordingly, i.e., quantization of this “wave” space utilizing denser grids results in better performance. On the other hand, increasing *K* diminishes the value of modeling itself—in the limit, grids start to become single points and the filter banks do not characterize global trends anymore; this drawback clearly resembles the overfitting phenomenon very well-known in machine learning. In summary, there is a clear multivariate trade-off between performance, number of dictionary atoms, computational load and risk of overfitting. Hence, it is still an open issue to select proper bounds on *K*; however, the empirical rules previously stated should be followed when possible.

Lastly, the proposed framework provides a richer parameter space to quantify neuromodulations. A set of EEP recordings can be characterized by its MPP features and timings, its filter bank properties, and the statistical moments of the noise component. Conversely, classical decomposition methods focus most of their attention on the power spectrum information alone and blur the temporal markers by introducing artificial processing windows. This is a consequence of the direct application of spectral estimation methods to non-stationary and transient signals that require special processing due to their particular generation mechanisms, noise properties and dynamical features. We have proposed a model flexible enough to be applied to event-related oscillations at different scales, topographical regions, and with diverse behavioral correlates, yet, specific enough to accommodate the particular properties required by the EEP. In addition, the methods can easily adjust to work in parallel with multiple trials, multiple channels, and multiple experimental tasks. In this way, the Marked Point Process quantification framework opens the door to novel analysis in deeper structures, such as the hippocampus and Deep Brain Stimulation-related targets, in the hope of elucidating novel mechanisms that can be correlated to the overt behavior and the microscopic activity thanks to the resulting precise temporal resolution.

In the spirit of openness and to encourage reproducibility, the MATLAB code corresponding to the proposed methods are available at https://github.com/cnel/MPP-EEG.

## Author contributions

CL, MO, and JP worked together and contributed equally to the methodology, validation, and preparation of the present paper.

### Conflict of interest statement

The authors declare that the research was conducted in the absence of any commercial or financial relationships that could be construed as a potential conflict of interest.
