# Supplementary material for: A Marked Point Process Framework for Extracellular Electrical Potentials
Source: Front Syst Neurosci. 2017 Dec 18;11:95. doi: 10.3389/fnsys.2017.00095 (PMC5741641; doi:10.3389/fnsys.2017.00095)
Supplement: Supplementary file 1 [file Table1.PDF]

Supplementary Material: A Marked Point Process Framework for Extracellular Electrical Potentials

1 SUPPLEMENTARY TABLES AND FIGURES

1.1 Tables

Relationship between average cross-correlation (electrode with maximum magnitude per finger) between Marked Point Processes obtained exploiting the proposed framework and finger movement kinematics with respect to the number of estimated dictionary atoms, K.

Table S1. Statistical significance of the average cross-correlation between smoothed high-gamma MPPs and finger flexion kinematics with respect to K. 1-way ANOVA. Subject 1.

	K							
Finger	2	5	12	25	50	75	100	p-value
1	0.32	0.34	0.35	0.41	0.39	0.41	0.41	0.38
2	0.30	0.40	0.44	0.45	0.44	0.44	0.43	0.26
3	0.03	0.08	0.14	0.17	0.15	0.15	0.17	0.49
4	0.23	0.26	0.30	0.32	0.34	0.35	0.34	0.52
5	0.21	0.28	0.30	0.31	0.31	0.31	0.33	0.86

Table S2. Statistical significance of the average cross-correlation between smoothed high-gamma MPPs and finger flexion kinematics with respect to K. 1-way ANOVA. Subject 3.

	K							
Finger	2	5	12	25	50	75	100	p-value
1	0.53	0.51	0.56	0.55	0.56	0.58	0.58	0.94
2	0.36	0.39	0.44	0.43	0.42	0.44	0.44	0.79
3	0.43	0.49	0.51	0.52	0.53	0.53	0.53	0.80
4	0.27	0.27	0.31	0.33	0.35	0.36	0.41	0.04
5	0.46	0.48	0.54	0.48	0.51	0.54	0.53	0.95
